# Management of a posterior tube exposure

**DOI:** 10.1016/j.ajoc.2024.102107

**Published:** 2024-07-23

**Authors:** Laura E. Barna, Lucy Q. Shen

**Affiliations:** Department of Ophthalmology, Massachusetts Eye and Ear, Harvard Medical School, Boston, MA, 02114, USA

Hello, this is Dr. Laura Barna, a glaucoma fellow at Mass Eye and Ear.

This is a case I performed with my attending, Dr. Lucy Shen.

It is a challenging tube revision of a very posterior exposure that is close to the plate.

This is a 70-year-old gentleman with a history of advanced POAG, who was noted to have a posterior tube exposure in his right eye. This is his third exposure of the **same** tube.

Here you see the surgeon's view of the eye showing the superotemporal quadrant.

The patient had significant trab scarring in the fellow eye before. Hence, an Ahmed valve was placed in this eye 7 years prior to presentation.

The first exposure occurred one month later at the limbus from conjunctival retraction and melting of the corneal patch graft.

The exposure was closed by tutoplast pericardium and advancing the conjunctiva.

5 years later, there was exposure of the anterior portion of the tube. At this time, he underwent cataract extraction, and the tube was rerouted to the sulcus and covered with tutoplast sclera.

Now he developed the third exposure of the posterior portion of the tube close to the plate. It was seidel negative and the patient was asymptomatic. The risk factors for this patient included dry eyes and large palpebral fissure.

Since the implant was originally placed, his visual field has been stable and his IOP has been controlled in the low teens.

We are posed with a difficult choice. Do we explant the tube, risking a loss of intraocular pressure control, move the tube to the pars plana, OR work with what we have and attempt to revise it. We decided to leave the tube in the sulcus and cover the exposed segment, as it is located posterior to the pars plana. We chose tutoplast sclera to cover the tube because this patient had a prior tube exposure with corneal patch graft.

With the eye infraducted, there is adequate exposure, and the area is cleaned with betadine. Lidocaine epinephrine is injected to elevate the surrounding conjunctiva. Using both blunt Wescott and vaness scissors, a gentle dissection is carried out.

Freeing the conjunctiva from any adhesions and scar tissue.

The limbus was not elevated, as the front segment of the tube was already covered adequately by tutoplast sclera from his previous tube revision surgeries.

Care was taken not to damage the tube during the conjunctival dissection.

Dissection was carefully performed over the capsule of the tube, making sure the conjunctiva was freed, without violating the tenon's capsule overlying the plate. This was carried out with Vaness scissors and blunt Westcott scissors. The adhesions between the conjunctiva and Tenons capsule were exposed and carefully dissected without creating buttonholes. My attending assisted me by clearing any blood to optimize visualization.

The dissection was carried out posteriorly to create a mobile flap of conjunctiva.

The dissection as also carried out anteriorly to ensure that the tutoplast graft can be tucked under the conjunctiva and be continuous with the previously placed tutoplast sclera.

This technique was used to prevent future tube exposure anterior to the repair site.

A piece of tutoplast sclera was brought onto the field and cut to the appropriate size.

This was sutured in place with two interrupted 9-0 VIcryl sutures on a spatulated needle.

The conjunctival flap was approximated and closed with several interrupted 9-0 vicryl sutures.

At post-op day 1 the patient's central vision was unchanged, the IOP was 16 and the patch was covered. He was started on moxifloxacin 4x per day for 1 week and prednisolone acetate 4x per day, which was then tapered weekly.

At post-op week 1 IOP is excellent at 14 on, latanoprost.

The tip of the tutoplast was exposed, but by POM1 this has epithelialized. At the most recent visit and 5 months since the revision, the tube remains covered.

We hope you will consider this technique when you are faced with recurrent tube erosion, particularly in an eye with well-controlled disease and IOP.

Thank you for your time and attention.

## CRediT authorship contribution statement

**Laura E. Barna:** Writing – original draft. **Lucy Q. Shen:** Writing – review & editing.

## Declaration of competing interest

The authors declare that they have no known competing financial interests or personal relationships that could have appeared to influence the work reported in this paper.

Acknowledgements

Dr. Shen received research support from the National Eye Institute (NIHR01EY031696), the Boston Keratoprosthesis Fund, and the Harvard Catalyst Fund (National Center for Advancing Translational Sciences, National Institutes of HealthAward UL1 TR002541). She is a consultant for FireCyte Therapeutics, Inc and AbbVie Inc.

